# An Infant's Question on COVID-19 and Music: Should I Attend My Online Classes?

**DOI:** 10.3389/fpsyg.2021.771050

**Published:** 2021-10-22

**Authors:** Efthymios Papatzikis

**Affiliations:** Department of Early Childhood Education and Care, Oslo Metropolitan University, Oslo, Norway

**Keywords:** infants (0 to 24 months), COVID-19, child development, distance learning, music, online behavior

## Abstract

In the last few months, we all have faced a profound challenge to balance our lives amidst fighting the COVID-19 pandemic. The reactions to this coronavirus pandemic have no doubt affected all aspects of our everyday normalcy as they have called for an extended set of measures that have greatly impacted our social interactions and well-being. During this unprecedented global situation, the pandemic has also taken its toll on education, as schools, universities, and other educational institutions have suspended their programs or moved online to retain educational momentum. Among the programs that tried to adapt to this online model was the early years music education. This mini-review article aims to discuss the framework of online existence for the early years music programs amid the COVID-19 crisis, while considering their benefits and character under these extraordinary circumstances.

## Introduction: The “COVID-19” Music Education Online Turn

Since the very beginning of the crisis, many people globally, turned to art and more specifically music in order to recharge, to discharge, to balance themselves and to feel supported. The first severe weeks of frustration and adaptation to this new “confined” reality brought a lot of examples of sing-along “balcony stages” and musical moments of relaxation and “distant” socialization in frontline working places like hospitals and caring homes.

Musical creativity, however, was not manifested only through casual everyday expressions or some new, reconsidered clinical approaches (Papatzikis et al., 2020). It continued flourishing in the context of the online education settings, too. Following the secondary and tertiary music education example into turning online (for e.g., see Calderón-Garrido and Gustems-Carnicer, 2021) many already established early-years music education programs tried to successfully accommodate parents/caregivers, “students” and specialist music educator-facilitators in this new online context by devising and offering relevant sessions. The mission and aims of these online sessions were to keep promoting development and further brain stimulation for children whose age ranges between a few months and 5 years (Gruhn, 2005); to propose early forms of sound and rhythm perception (Papatzikis and Papatziki, 2016) among other musical qualities; to offer pre-lexical or early speech communication platforms (Bolduc, 2009; Walton, 2014), but most of all to support and facilitate social interactions, development and bonding (Hallam and Council, 2015). These have always been the goals of early music programs but for the first time they were taking place in a synchronous—sometimes asynchronous too—online educational context approached mostly as “emergency remote” rather than “online” teaching (Hodges et al., 2020).

## The Reality of This Online Turn

Many early-years music educators embraced this new emergency remote teaching framework and found a platform upon which they continued offering their services and passion for music during the crisis. Moreover, many parents and caregivers happily endorsed the initiative, realizing the potential of extending social interaction as well as the development and shades of normalcy opportunities for their confined infants and toddlers.

Given the circumstances, however, many quite known (McPake et al., 2013) challenges emerged as a result of the virtual interactions. Poor quality of sound; blurred or time-delayed video-stream; overcrowded and pluralistic land- and soundscapes which offensively and randomly merge acoustic and visual stimuli coming simultaneously from the music facilitators and the attendees' immediate surroundings, were some of the elements that started creating problems to the sessions' design and their learning outcomes (Kim, 2020). Many motivation obstacles also seemed to emerge (Martínez-Castilla et al., 2021) when both educators and caregivers realized that non-physical interaction prevails—as of course is to be expected in this online context—producing a fragmented reality of the previously established “communicative musicality” and musical interaction. The fairly calm, structured and controlled physical context of the on-site early-years music education sessions had to be now supplanted by a number of physical and emotional distractors emanating from unavoidable COVID-19 related elements and sources. Ultimately, the mental load needed to participate started becoming more demanding than before (Galea et al., 2020). It skyrocketed for all participants—even the youngest ones—as they embarked to fight screen fatigue, multitasking, the disrupted audio-visual queues, and the false perhaps sense of diminished attentiveness and conscientiousness between the interlocutors; all common mental load denominators found in every context of distance learning (Schoenenberg et al., 2014).

## Why Continue With the Online Early-Years Music Education?

Such a non-favorable online reality might eventually bring some quite negative appraisals of the early-years music education initiatives. It might start pushing away interested caregivers who believe that effectiveness in this context solely relies on the music facilitator's extensive skills and immediate interaction with their children; a quality that is not directly available through this mode of session delivery. It might also make some of the caregivers doubt their positive involvement, not having in place anymore the immediate, physical support of the music facilitator. At the same time, considering the physical distance, music educators might start feeling that they do not convey “the message” properly.

To eventually avoid the negative impact the COVID-19 pandemic can bring to this specific educational field, it might be important to highlight some major elements that can still pertain to the online early-years music education context, making it therefore a valuable educational and socialization alternative to this or other similar crises.

### Music Socialization Can Still Happen Online

Music leads us to socially connect through parallel and synchronous movement and body entrainment (Merker et al., 2009; Knoblich et al., 2011). Imagine people singing the same song all together. The ensemble of singers becomes a synchronous system of physical movement either via dancing, or moving their hands, or even via moving their body-core. They all start feeling the same rhythm. Studies related to the bio-mechanic character of music's impact on humans have shown that many of our body and brain parts manage to perfectly synchronize (Müller and Lindenberger, 2011; Greenberg et al., 2021). Such a synchronization makes us feel socially present and active (Wiltermuth and Heath, 2009; Good et al., 2017) while also help us ease pain, increase its threshold and “fight” psychological discomfort (Weinstein et al., 2016). Considering (a) that a synchronous music movement and body entrainment can indeed take place at a certain extend during the online interaction, as well as that (b) studies on online synchronous interaction have suggested that social presence can be maintained in this context (Cobb, 2009) we understand that perhaps a far-fetched, yet possible form of socialization can still emerge and sustained through the particular mode of delivery.

### Mentally, Online Sessions May Be Better Than No Sessions at All

Research at a sociobiological level has repeatedly shown that musical interaction promotes social connectionism (Hove and Risen, 2009; Chanda and Levitin, 2013) and social adaptation (Tarr et al., 2014) by helping our bodies adjust their reaction to their environment. More specifically, it has been found that social singing can decrease the levels of cortisol (a stress biomarker), it can increase the oxytocin levels in our bodies (a biomarker of social bonding), while it can also increase the levels of β-endorphins (a biomarker connected to reward and pain thresholds) (Kreutz, 2014; Fancourt et al., 2016; Weinstein et al., 2016). Moreover, neuroscientific studies (Fasano et al., 2019; Klepzig et al., 2019; Martínez-Molina et al., 2019; Nemati et al., 2019; Shany et al., 2019; Greenberg et al., 2021) have also suggested that (social) music engagement may well-increase our brain activation, the feeling of well-being as well as the levels of our focus and attention. Considering the severe impact the COVID-19 confinement measures may have on our mental health (Holt-Lunstad et al., 2010; Galea et al., 2020) as well as the toll a potentially socially deprived environment might take on infants' and toddlers' brain (Innocenti, 2007), it seems important to continue offering these sessions even if not in their best available form.

### The Online Reality May Benefit the Infant-Parent Dyad Bonding

Early-years music sessions are greatly valued from many parents and caregivers because they offer an extensive platform for early social interactions. They promote parent and child learning while establishing connections within and between different families (Rodriguez, 2019). Children in the early music education context learn to perceive communication via a triadic system (i.e., self-parent- “third party”) while they create social partnerships of equal and cooperative members through entrainment, social/emotional referencing, joint attention and joint action (Ilari, 2016). Nevertheless, in this new online context, it is quite evident—from its technical requirements and framework (Qasmi et al., 2021)—that while entrainment and joint action can somewhat be achieved for the triadic system, this cannot easily happen for social/emotional referencing and joint attention. Emotional referencing and alignment with a third party, outside of the parent-infant dyad, seems very difficult to get achieved for technical reasons. The same applies to joint attention; especially for the younger participants who may not be able to perceive the dynamics and properties of the online communication context. As a result, children may be found more distracted, energy drained, or even non-attracted at all by the specific learning process. Despite these challenges, however, their realization could illuminate a positive path to follow and benefit from. The infant-parent dyad could enhance their interactions repertoire and provide more time and space for the dyadic system to flourish as proven in previous research (Niedzwiecka et al., 2018; Corkin et al., 2021). Additionally, more opportunities may arise for the parents to explore and refine their own involvement in musical and communication terms (i.e., invest more into trying signing with their children; experimenting and furthering techniques of musical interaction already known from previous physical sessions etc.).

## Discussion

There is no doubt that the online (a)synchronous early-years music engagement synthesizes a demanding educational environment. This environment showcases a great list of both negative and positive points to consider. It is a new delivery mode introducing unchartered waters for both parents and educators. Admittedly, this mode of delivery can in principle be very helpful. However, it came unfortunately in use at a difficult time, amidst a pandemic, where emotions, practices and results are greatly tested and stretched.

A first reaction to this abrupt online turn would be to consider that families should take away with them whatever they can handle and are happy with. Therefore, it might be helpful to remind them that it does not have to be all about the infants and toddlers engaging with the facilitators via the screen. Parents should look for engaging more actively with their infants, appraising even more so the involvement of the “online” facilitator as guidance for them rather their children. Afterall, the parents should be the major catalysts in the educational and developmental process in the early years; be it either online or offline. Parents might even need to guide professional practitioners to more efficient and reliable communication techniques in this demanding context. In the end, more field research is definitely needed to start mapping and translating this complex yet promising for current and future applications online mode of early-years music engagement. The discussion interconnecting music, education, mental health and COVID-19 may have come here to stay for a while longer as relevant research shows (Mastnak, 2020) and we therefore owe to offer to the youngest ones a well-informed answer on whether or not (and how) to better attend online early years music education classes.

## Author Contributions

EP conceived, wrote, and edited the manuscript.

## Conflict of Interest

The author declares that the research was conducted in the absence of any commercial or financial relationships that could be construed as a potential conflict of interest.

## Publisher's Note

All claims expressed in this article are solely those of the authors and do not necessarily represent those of their affiliated organizations, or those of the publisher, the editors and the reviewers. Any product that may be evaluated in this article, or claim that may be made by its manufacturer, is not guaranteed or endorsed by the publisher.
